# Supplementary figures and images for: Antifungal Combination Eye Drops for Fungal Keratitis Treatment
Source: Pharmaceutics. 2022 Dec 22;15(1):35. doi: 10.3390/pharmaceutics15010035 (PMC9866460; doi:10.3390/pharmaceutics15010035)

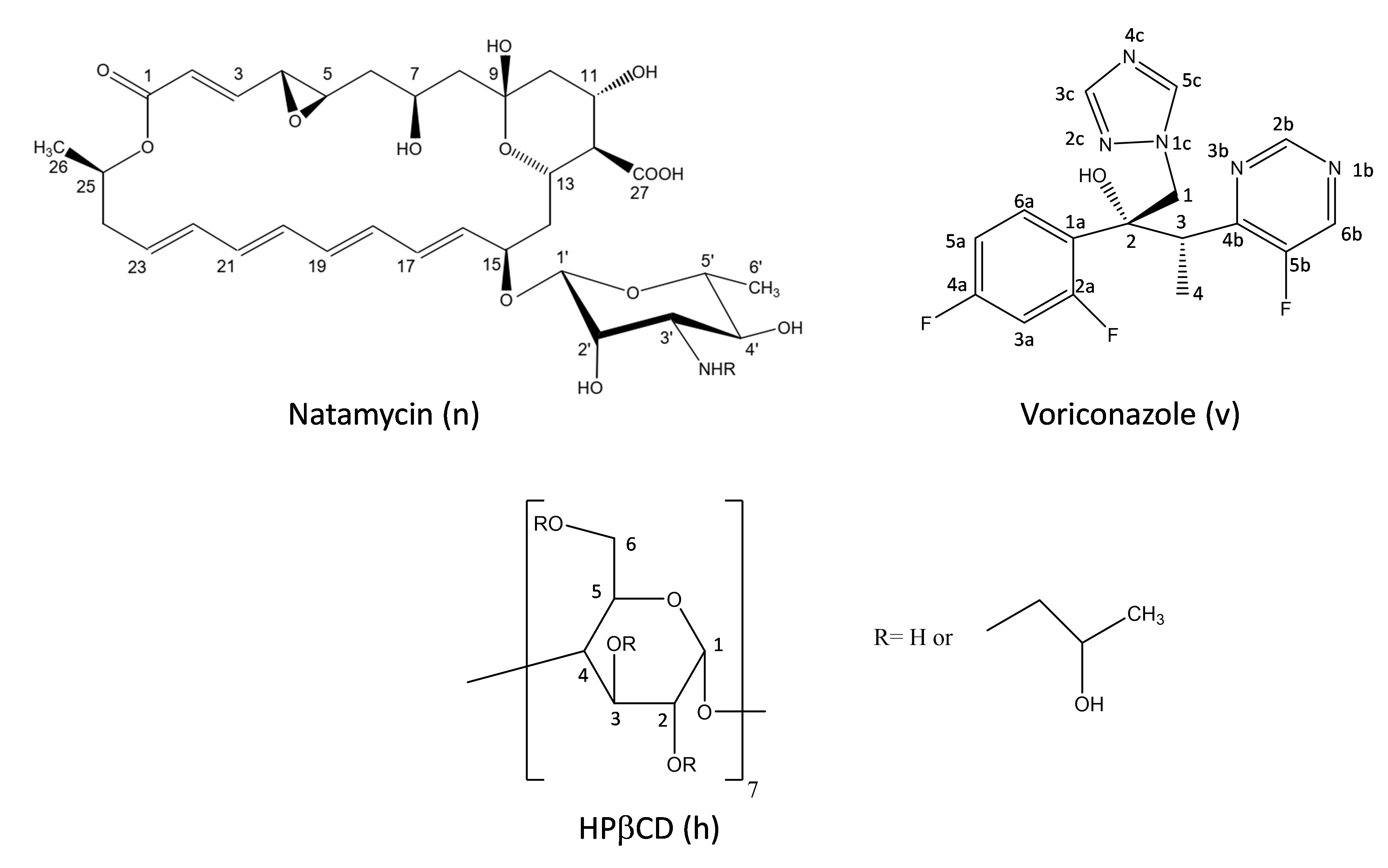

Supplement: Supplementary file 1 [file pharmaceutics-15-00035-s001.zip › figure S1.jpg]

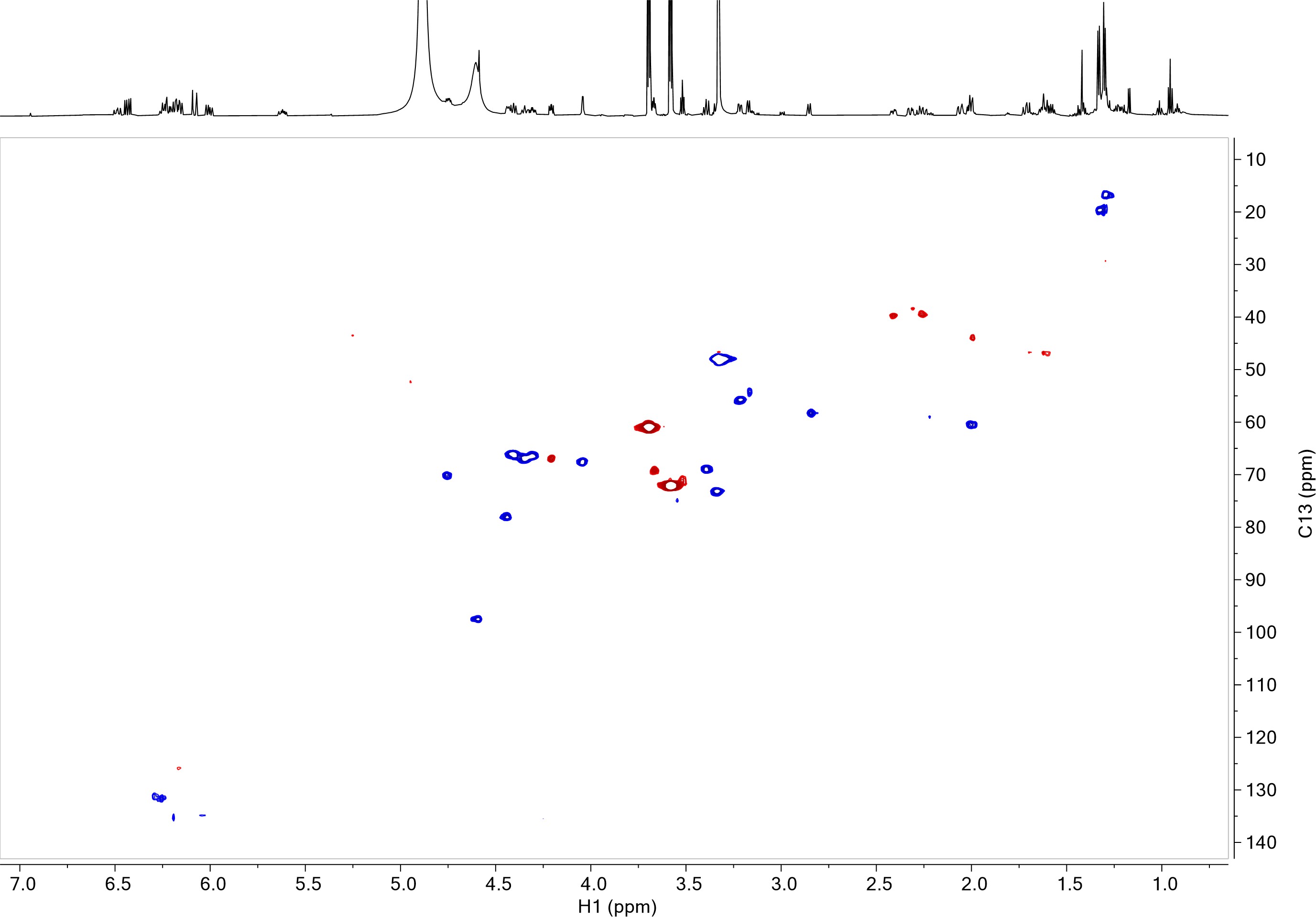

Supplement: Supplementary file 1 [file pharmaceutics-15-00035-s001.zip › figure s2.jpg]

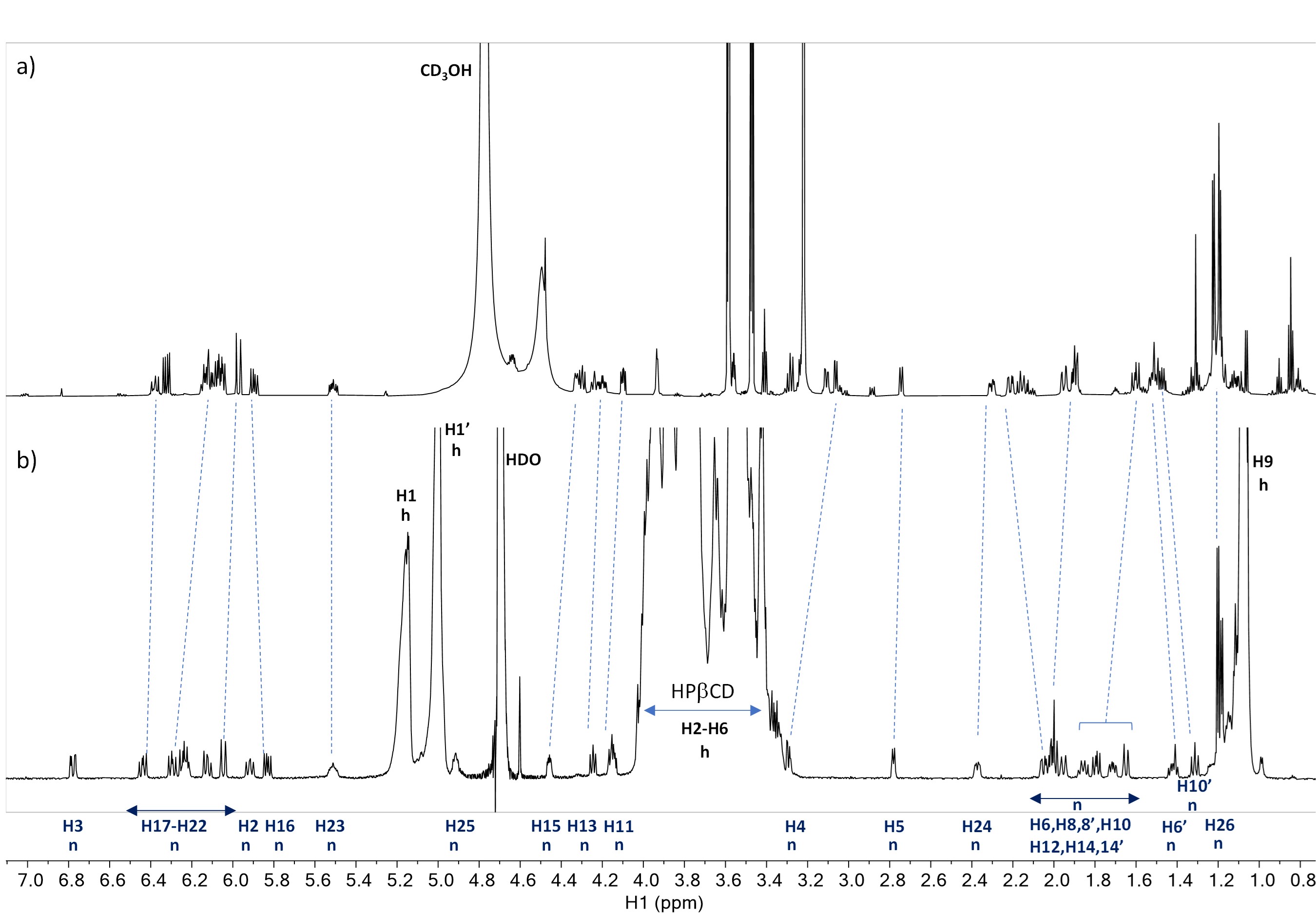

Supplement: Supplementary file 1 [file pharmaceutics-15-00035-s001.zip › figure s3.jpg]

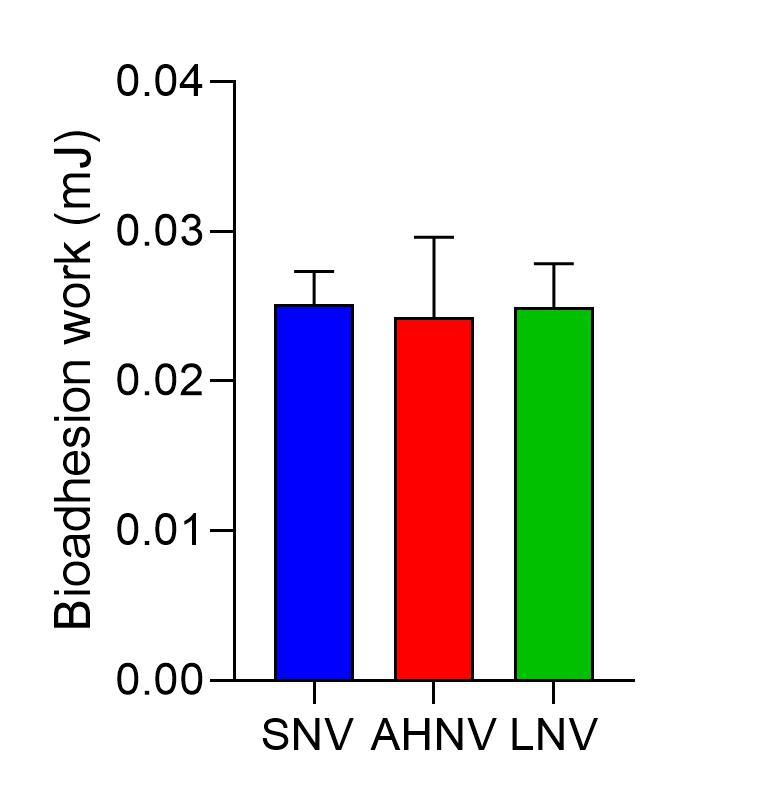

Supplement: Supplementary file 1 [file pharmaceutics-15-00035-s001.zip › figure s4.jpg]

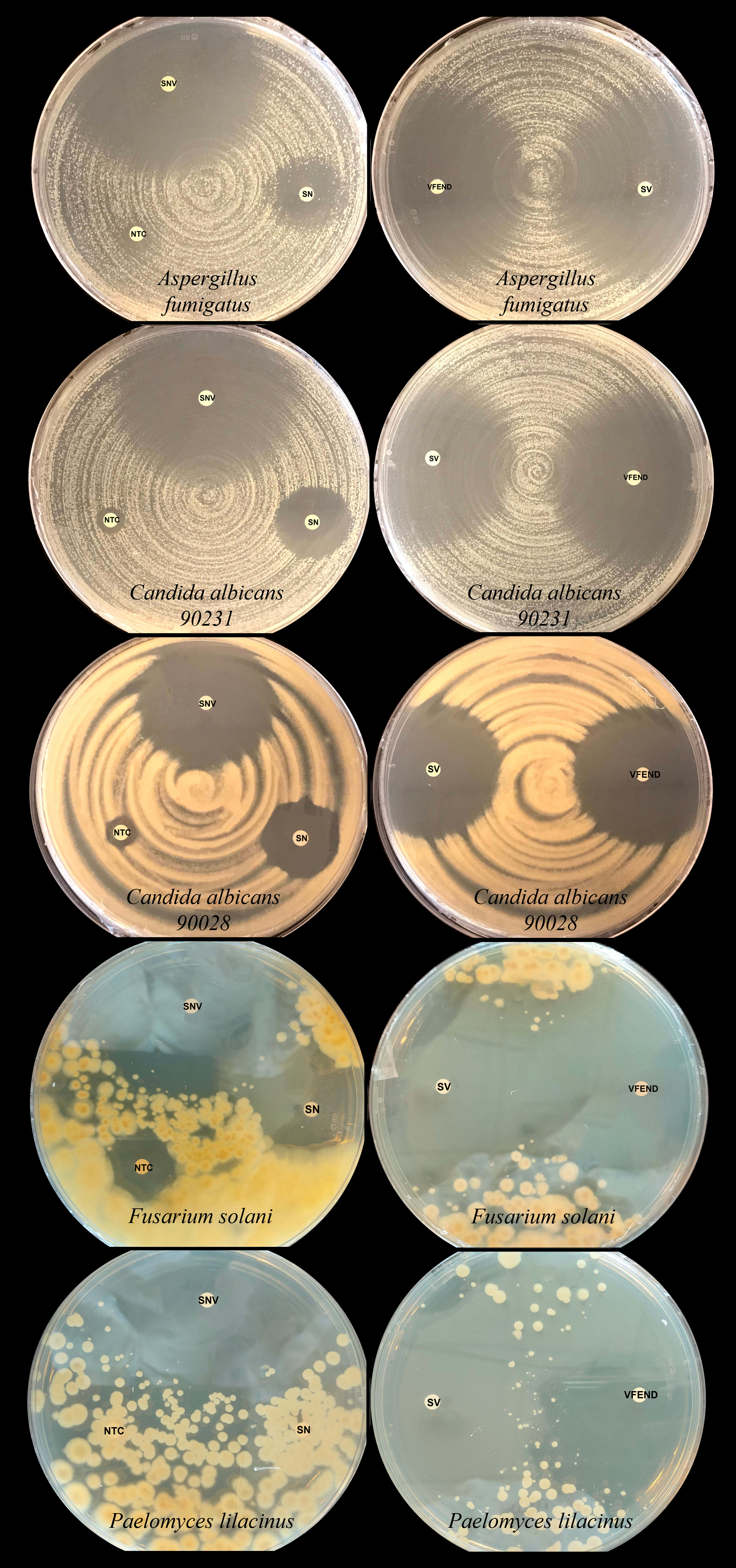

Supplement: Supplementary file 1 [file pharmaceutics-15-00035-s001.zip › figure S5.jpg]

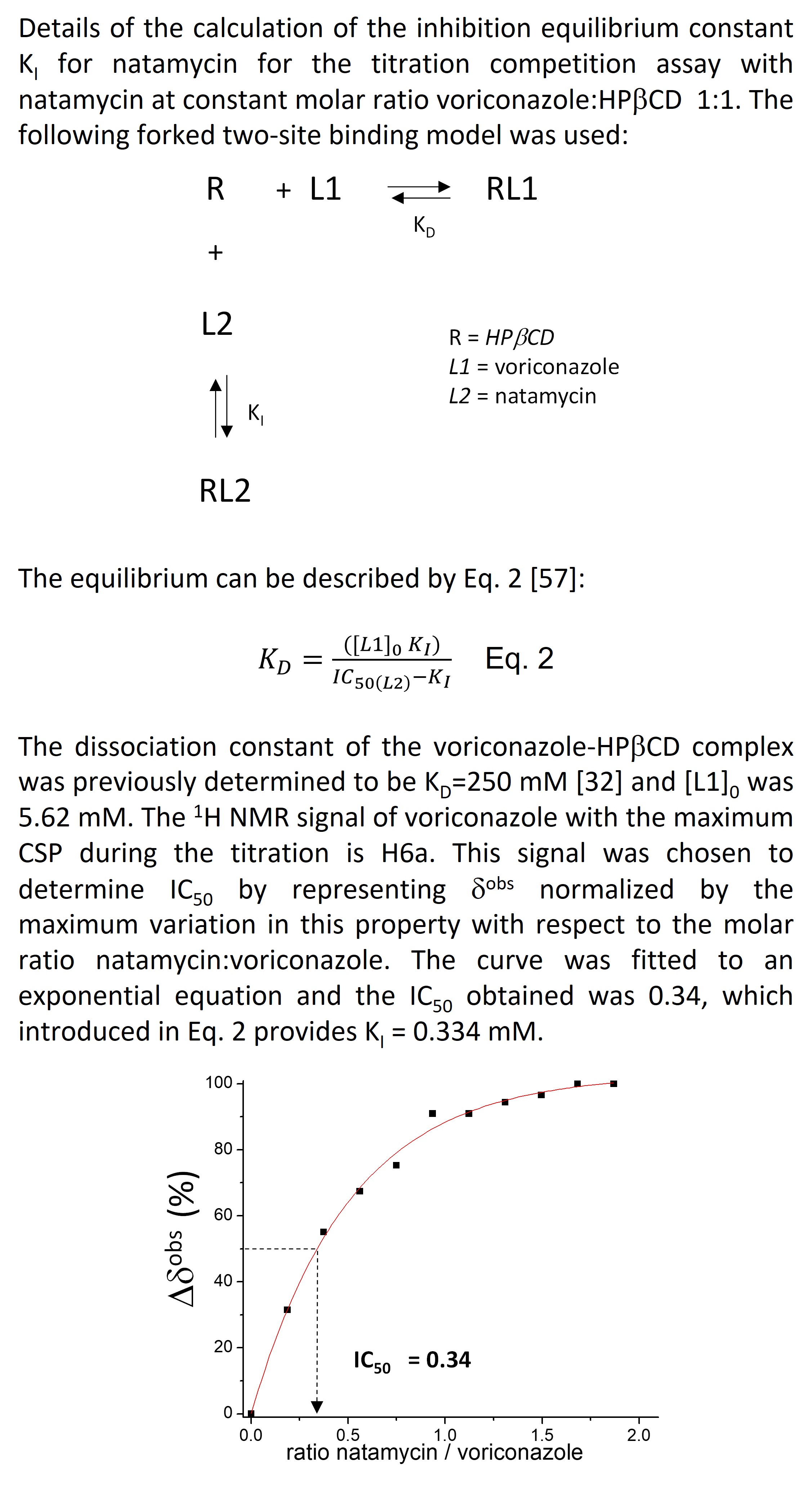

Supplement: Supplementary file 1 [file pharmaceutics-15-00035-s001.zip › sup. calculation.jpg]
